# First archaeological evidence for ginger consumption as a potential medicinal ingredient in a late medieval leprosarium at St Leonard, Peterborough, England

**DOI:** 10.1038/s41598-024-52422-8

**Published:** 2024-01-30

**Authors:** Elena Fiorin, Charlotte A. Roberts, Marica Baldoni, Erin Connelly, Christina Lee, Claudio Ottoni, Emanuela Cristiani

**Affiliations:** 1 Department of Oral and Maxillofacial Sciences, DANTE—Diet and Ancient Technology Laboratory, Sapienza University of Rome, 00161 Rome, Italy; 2 Department of Archaeology, Durham University, Durham, DH1 3LE United Kingdom; 3 Centre of Molecular Anthropology for Ancient DNA Studies, Department of Biology, University of Rome Tor Vergata, 00133 Rome, Italy; 4 School of Life Sciences, University of Warwick, Coventry, CV4 7AL United Kingdom; 5 School of English, University of Nottingham, Nottingham, NG7 2RD United Kingdom

**Keywords:** Infectious diseases, Ecology

## Abstract

Leprosy was one of the most outwardly visible diseases in the European Middle Ages, a period during which leprosaria were founded to provide space for the sick. The extant documentary evidence for leprosy hospitals, especially in relation to diet, therapeutic, and medical care, is limited. However, human dental calculus stands to be an important source of information as it provides insight into the substances people were exposed to and accumulated in their bodies during their lives. In the present study, microremains and DNA were analysed from the calculus of individuals buried in the late medieval cemetery of St Leonard, a leprosarium located in Peterborough, England. The results show the presence of ginger (*Zingiber officinale*), a culinary and medicinal ingredient, as well as evidence of consumption of cereals and legumes. This research suggests that affected individuals consumed ingredients mentioned in medieval medical textbooks that were used to treat regions of the body typically impacted by leprosy. To the authors' knowledge, this is the first study which has identified *Zingiber officinale* in human dental calculus in England or on the wider European continent.

## Introduction

Leprosy, or Hansen’s disease, is a chronic bacterial disease with a variety of risk factors that predispose people to this infection. It is still present in some parts of the world today but has been declining for many years^1^. It is caused by either *Mycobacterium leprae* or the more recently identified *Mycobacterium lepromatosis.* Inhalation of bacteria-laden droplets is the most accepted method of transmission of leprosy from human to human. Furthermore, prolonged and close contact with an infected person over weeks or months is necessary for the transmission of leprosy^2^. During the incubation period, which usually ranges from 2–10 years or more, a person may not display any signs or experience any symptoms. The disease affects the peripheral nerves, the skin, the upper respiratory tract, and other parts of the body, such as the skeleton. In addition, people with leprosy may develop mental health challenges, which are caused in part by social stigma^3^. Bioarchaeologists are able to recognise leprosy in archaeological human remains from skeletal changes in the cranial and postcranial bones (e.g.,^4–6^). Indications include the absorption and remodelling of the nasal aperture, absorption and recession of the bone of the anterior part of the upper jaw, inflammatory changes to the palate, and loss of the anterior teeth (rhinomaxillary syndrome). The leprosy bacteria can damage the sensory, motor and autonomic nerves, which can lead to ulceration of the hands and feet, flexion deformities of the fingers and toes, and ultimately potential damage to bones. Diagnosis of leprosy relies on bone changes of the facial, hand and foot bones, but those of the facial bones are most characteristic and specific for leprosy^5^.

From the end of the eleventh century to the dissolution of the monasteries in England in the sixteenth century, over 300 leprosy hospitals were founded to provide a space for the sick^7^, often by benefactors to guarantee the ‘speedy passage through the fires of purgatory’(^8^, p.106). From the fourteenth century, the number of hospitals declined. This may be because of a reduction in the number of infected people but also due to other reasons, such as high mortality during the Black Death, including in people with leprosy, an increase in tuberculosis, or the impact of mitigative measures at leprosaria themselves^9^. Furthermore, while surviving account books and other documents related to leprosaria may provide useful information about care provision and other aspects of daily life, they do not always contain explicit information about treatments, specific medicines, or the patient experience^5^. That is why direct scientific evidence from human remains is important. The analysis of skeletal remains from medieval hospital cemeteries is a source of information for the substances an individual was exposed to and which accumulated in their bodies during their life. In addition, dental calculus analysis is well-positioned for identifying plants (e.g., herbal remedies), metals, and other possible medicinal ingredients.

Human dental calculus is composed of inorganic (calcium phosphate salts) and organic (lipids, carbohydrates, DNA molecules and proteins) components^10^. Its accumulation on teeth, as well as its composition and quantity, varies between people and is influenced by factors such as oral hygiene, diet, age, genetic profile, and diseases^11^. The analysis of ancient calculus has proved to be a highly promising and informative analytical technique in bioarchaeology because calculus traps and preserves particles of food and other ‘materials’ ingested or inhaled during the individual’s life (e.g.,^12–15^). These analyses also permit the identification of plant species, which could be reflective of medicinal remedies rather than food consumption (e.g.,^16–18^). As a record of ancient biomolecules, dental calculus has also proved to be an invaluable tool for investigating ancient oral microbiomes via shotgun metagenomics^19^. A recent study succeeded in reconstructing the full genome of the bacterial etiological agent of leprosy, *Mycobacterium leprae,* from dental calculus. This suggested that DNA molecules of the microorganism were incorporated into the mineral matrix of dental calculus via lesions of early-stage leprosy in the oral cavity^20^. Furthermore, antimicrobial resistance (AMR) is present in host-associated oral microbiomes, and metagenomic analysis of ancient dental calculus recently demonstrated that it occurs in natural environments and in ancient human and animal samples^21,22^.

**Figure 1:**
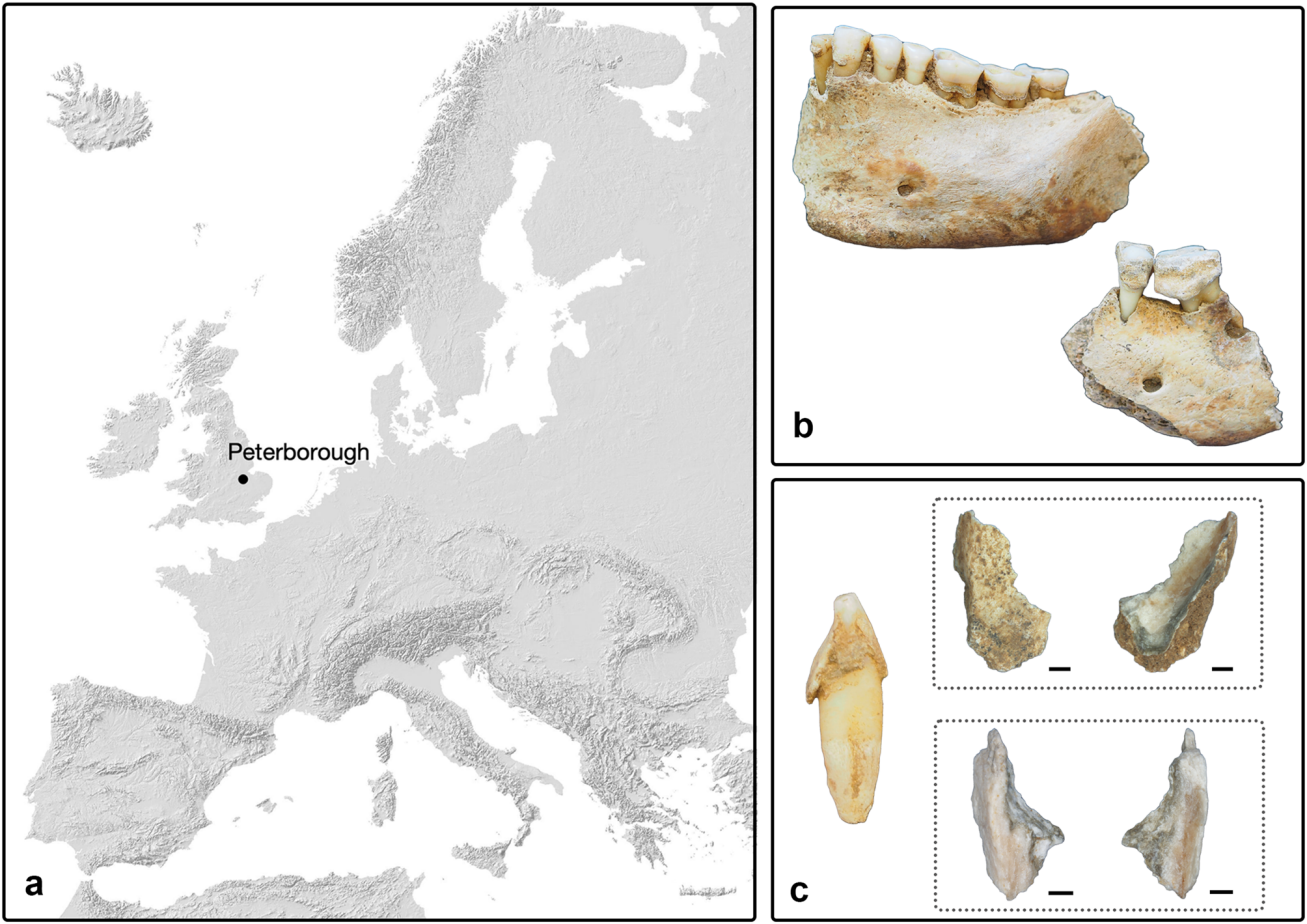
(**a**) map of Europe showing Peterborough’s location; (**b**) dental calculus quantity and distribution among the individuals (P32 on the left, P33); (**c**) an example of the results obtained following the cleaning process. On the left, the lower right second incisor of an adult female (P91) before sampling. The upper box shows the external and internal sides of the calculus photographed with a high-resolution stereo microscope following sampling. Soil deposits are clearly visible. The lower box shows the same fragment of calculus (external and internal sides) after manual cleaning. Scale bars are 1000 μm.

The aim of this research was to use dental calculus analysis to add new information on the medical treatment of leprosy and, in particular, the medicines offered to individuals who were living in a leprosarium in the late medieval period. This study performed analyses of individuals buried in the cemetery linked to St Leonard, a medieval leprosarium located in Peterborough, England (Fig. 1a). No original hospital buildings have survived, and little historical information about such structures is available^23^. The results obtained by employing polarised light microscopy revealed dietary behaviours and the use of potential medicinal ingredients in treating individuals affected by leprosy. Furthermore, to screen the samples for the presence of *M. leprae* DNA molecules and test whether the disease and the daily life of the people in the leprosarium affected the human oral microbiome to any degree, metagenomic analysis of four individuals showing macroscopic skeletal evidence of leprosy (i.e., rhinomaxillary syndrome) was conducted.

## Results

### Polarised light microscopy analysis

Dental calculus was analysed from 42 skeletons of the 130 individuals excavated from two chronological phases of the cemetery associated with the St Leonard leprosarium^24^. Microremains were found in the calculus of 40 of the 42 individuals (Dataset S1). Note that the number of findings does not correlate with the number of slides prepared or the weight of the samples.

**Figure 2:**
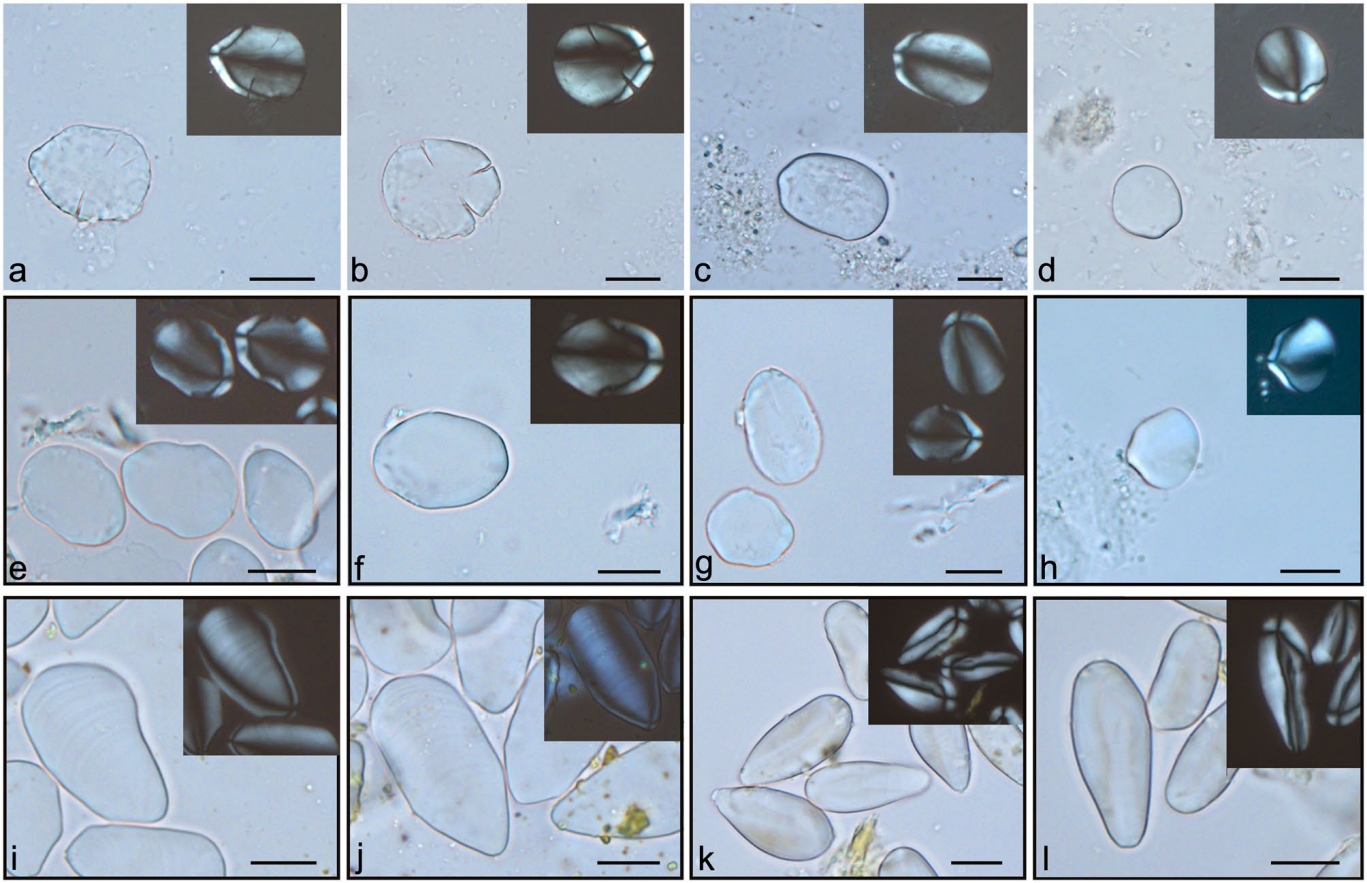
Starch grains identified as *Zingiber officinale* in dental calculus from the St Leonard individuals and experimental reference (black-framed photos). Scale bars are 20 μm. (**a**, **b**) Partially damaged starch grains from the calculus of the indeterminate adult buried in grave 35 who shows possible skeletal evidence of leprosy; (**c**) Starch grain from the calculus deposit of the adult female buried in grave 85 who shows skeletal evidence of lepromatous leprosy; (**d**) Starch grain from the adult female buried in grave 45 who shows skeletal evidence of lepromatous leprosy; (**e**, **f**, **g**) Modern ovoid-elongated bell-shaped starch grains of ginger (*Zingiber officinale*). Notice the ovoid-elongated bell shape and the eccentric hilum. Lamellae are barely visible. The features are similar to those found in the individuals buried in graves 35 and 85; (**h**) Modern starch grain of ginger (*Zingiber officinale*) after processing. The modern tuber was cut into small pieces, boiled for 5 min in ultrapure water, left in the infusion for another 5 min and observed under the microscope. Subsequently, the infusion was stored in the fridge for 6 months and observed again at the microscope. Evidence of intact starch grains was observed in both cases. This is one of the starch grains retrieved from the six-month infusion. Note that the starch is still intact and the morphology is similar to the starch grain observed in Figure d; (**i**, **j**) Modern starch grains of Curcuma (*Curcuma longa)* characterised by an elongated-triangular shape, clearly visible lamellae, and an extremely eccentric hilum; (**k**, **l**) Modern starch grains of galanga (*Alpinia officinarum)* characterised by a very elongated-lanceolate and a narrow morphology.

Starch grains, produced by most green plants as a form of energy storage, were the main finding. They were present in 69% of the individuals (N = 29) and more abundant in Phase I (N =  > 159, Phase I; N = 30, Phase II). Starch grains with diagnostic features for taxonomic identification were classified into five morphotypes (see *SI Appendix f*or morphotype descriptions, Dataset S1 and S2, Figure S1). **Type I** (Triticeae tribe) was observed in eight individuals from Phase I (two males and six females) and six from Phase II (five males and one female) (Figures S1a, S1b, and S1c). **Type II** (Panicoideae subfamily) was observed in seven individuals from Phase I (three males and four females) and three from Phase II (two males and one female) (Figs. S1h and S1i). **Type III** (Fabaceae) was detected in two individuals from Phase I (two females) and two from Phase II (two males) (Figs. S1k, S1l and S1m). **Type IV** was observed in seven individuals from Phase I (Fig. 2a–d). Six were female individuals, and one was indeterminate. No individuals from Phase II had evidence of Type IV starch grains. According to the literature, the starch grains identified belong to the *Zingiber* sp*.* of the Zingiberaceae family^25–27^. Through comparison with the modern reference collection created for this study, these grains were attributed to *Zingiber officinale*, excluding those belonging to *Curcuma longa* and *Alpinia officinarum* which also belong to Zingiberaceae and were present in late medieval Europe^28^ (Table 1, Fig. 2, see also *SI Appendix*). Since *Zingiber* sp. starch grains have a diagnostic morphology, the modern reference collection used to compare modern and archaeological starch grains confirmed the identification with ginger. Figure 2 shows that the dimensions and morphology of the starch grains observed in the St Leonard individuals are compatible with those of *Zingiber officinale.* Moreover, modern ginger starch grains were processed (boiled and left in infusion) to test their preservation qualities (Fig. 2h). This experiment shows that the starch grains survived and preserved their features. This indicates that ginger starch grains could have entered the mouth and thus become deposited in the calculus by ingestion of both raw tuber and processed food or drinks. **Type V** (Fagaceae) was observed only in two individuals from Phase I (two females). Other starch grains were not specifically identified because they were damaged (N = 12, both phases) or non-diagnostic (N = 26, both phases) (Dataset S2).

**Table 1:** Description and summary statistics of the starch grain lengths (μm) and widths (μm) of *Alpinia officinarum*, *Curcuma longa*, and *Zingiber officinale*.

Species	N	Length						Width					
		Mean	Median	Max	Min	SD	IQR	Mean	Median	Max	Min	SD	IQR
*Alpinia officinarum*	165	43.90	45.31	65.77	16.50	10.18	12.69	19.94	19.55	29.11	12.34	2.92	4.70
Starch grain morphology: elongated-lanceolate shape. The hilum is eccentric. Lamellae are present but barely visible. The main axis (maximum length) ranges between 16.50 and 65.77 μm (mean size of 43.90 μm) and the maximum width ranges between 12.34 and 29.11 μm (mean size of 19.94 μm)													
*Curcuma longa*	165	53.65	52.98	85.48	26.28	10.01	11.85	32.81	31.21	68.21	21.69	7.58	5.38
Starch grain morphology: elongated-triangular shape. The hilum is extremely eccentric and narrower near the hilum. Lamellae are clearly visible. The main axis (maximum length) ranges between 26.28 and 85.48 μm (mean size of 53.65 μm) and the maximum width ranges between 21.69 and 68.21 μm (mean size of 32.81 μm)													
*Zingiber officinale*	165	35.04	35.66	54.68	11.64	7.30	7.80	25.02	25.91	34.86	10.11	5.13	5.82
Starch grain morphology: ovoid-elongated bell shape with an eccentric hilum usually towards the narrow end. Lamellae are barely visible. The main axis (maximum length) ranges between 11.64 and 54.68 μm (mean size of 35.04 μm) and the maximum width ranges between 10.11 and 34.86 μm (mean size of 25.02 μm)													

Fragments of vegetal remains (i.e., tissues, fibers, and vascular elements) were present in (again) 69% of the population (Dataset S1, N = 29 individuals). None of these microremains showed diagnostic features for specific identification, and they were mostly concentrated in three individuals: an adult female from Phase I (P42, N =  > 680, *SI Appendix*, Figs. S2e and S2f.), and an adult female and male from Phase II (P83, N =  > 56; P79, N =  > 30). Comparing the vegetal remains found in P42, similarities with those observed in the spikelets of *Lolium arundinaceum* were observed (Poaceae family, *SI Appendix*, Figs. S2g and S2h). Phytoliths were found in two female individuals from Phase I (N =  > 8, P42 and P55) and in one male individual from Phase II (P120, N =  > 9) (Dataset S1). Phytoliths of Phase I are represented by multi-cell phytoliths composed of long cells with clavate/sinuate margins^29^. These morphologies are compatible with those found in grasses (Poaceae)^30,31^. In Phase II, several multi-cell long-cell phytoliths were observed embedded in the calculus matrix, but they were not identified because they were not clearly visible. Only two pollen grains were found in Phase I (Dataset S1).

Amongst the microremains of animal origin, barbules (N = 2, Phase I), fungal spores (N = 11, Phase I; N = 18, Phase II) and fragments of insect remains, all of which were hairs (N = 12, Phase I; N =  > 49, Phase II) were observed. Except for a fragment that could be from a bee hair, the other hair fragments might belong to insects of the Dermestidae family^32,33^ (Dataset S1, *SI Appendix*, Figure S2a). The hairs were mostly still embedded in the calculus, and they were found in three males and six females from Phase I and four males and one female from Phase II (Figs. S2b and S2c). In an adult female from Phase 2 (P83), hair fragments of the dermestid were found mixed with several vegetal remains (i.e., tissues, fibers, and vascular elements) and fungal spores (P83, N =  > 42, *SI Appendix*, Fig. S2d). The other remains were sparse, comprising small fragments of charcoal (N = 9, Phase I; N = 10, Phase II) and unidentified elements (N = 11, Phase I; N = 4, Phase II) (Dataset S1). They are non-diagnostic, and their origin is difficult to establish.

### Ancient DNA analysis

#### DNA preservation

Using shotgun sequencing, between 25 and 44 million sequences were generated for four genomic libraries constructed from the DNA extracts of the dental calculus of four individuals, two adult males (P18 and P68) and two adult females (P45 and P112). After adapter trimming and quality filtering, 33 to 52% of the reads could be assigned to Bacteria and Archaea by interrogating a custom database of complete microbial genomes from the NCBI RefSeq and GenBank with Kraken2^34^ (*SI Appendix,* Table S1). More than 88% of the reads classified at the species level were assessed as deriving from the oral environment with Sourcetracker (*SI Appendix,* Figure S5, Table S2). The authenticity of our data was further demonstrated by the high terminal deamination pattern detected after aligning the reads of each sample to the reference genome of the most abundant Bacterial species detected with Kraken2 (Dataset S3).

#### Taxonomic analysis

Taxonomic classification with Kraken2 assigned < 0.001% of classified reads (up to 24 reads, sample P45) to *M. leprae*, significantly less than the value (4.5% of classified reads) observed in a previous study of ancient dental calculus from an individual affected by early stage leprosy^20^*.* Furthermore, these reads most likely originated from a few small segments of the reference genome (*SI Appendix*), and therefore represent false positives. This result was confirmed by screening the sequencing data with MetaPhlAn3^35^, which did not detect *M. leprae* (Dataset S5). To characterise the oral microbiomes reconstructed in the four individuals analysed, oral microbial variation at the species level was contrasted against other microbiome datasets that were available from the published literature (Dataset S6). In particular, human dental calculus data derived from samples from medieval Ireland^36^ and eighteenth–nineteenth century England^37^. Present-day plaque and dental calculus datasets were also included for comparative purposes^37,38^.

**Figure 3:**
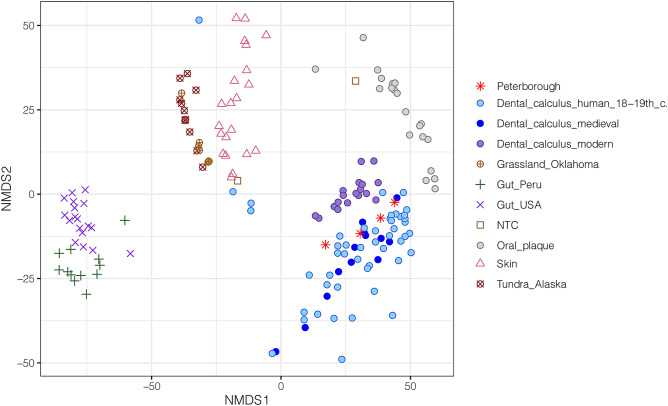
Non-metric Multidimensional scaling of Aitchison distances of bacterial and archaeal species abundances. Dental calculus samples from St Leonard (Peterborough) were plotted along with other oral, skin, gut, soil, and laboratory negative controls (NTC) microbiomes.

Microbial variation in the comparative dataset was first explored with a non-metric multidimensional scaling (nMDS) of Aitchison distance estimates from species abundances (Fig. 3). The results showed that the four individuals from the St Leonard leprosarium clustered together with the medieval and eighteenth–nineteenth century samples from Ireland and England, respectively. Significant differences in the microbial composition of the groups investigated were tested through permutational multivariate analysis (Permanova) of CLR-transformed Euclidean distances calculated from microbial species abundances. While the modern dental calculus sample appeared to be different (*P* = 0.006), no significant changes were found when contrasting the historical samples (PB *vs*. IRE *P* = 0.156; PB *vs*. UK *P* = 0.294, adonis test) (*SI Appendix,* Tables S3, S4).

Potential differences in species abundances of the samples investigated were tested with DESeq2. While the individuals from St Leonard showed a relatively high number of differentially abundant species (> 20) when compared with the modern dental calculus sample and eighteenth-nineteenth century sample from England, only one species was found when compared with the medieval Irish data (*Actinomyces gerencseriae*), thus suggesting an overall homogeneity at the taxonomic level between the microbiomes of the two samples investigated (*SI Appendix,* Fig. S6). However, the comparative dataset that was used is very limited by the availability of samples so far investigated and published in the literature. Further metagenomic analyses on dental calculus from medieval England will help to assess potential changes in oral microbiome compositions more directly in the future.

#### Antimicrobial resistance (AMR)

To investigate AMR activity in the oral microbiota of the individuals analysed, potential changes in the AMR gene family abundances of the St Leonard dental calculus samples were screened against the comparative dataset. The Basic Local Alignment Search Tool (BLAST) and the Comprehensive Antibiotic Resistance Database (CARD) were used to find the number of reads matching AMR gene families, as previously described^21^. The analysis showed that reads mapping the *cfxA *beta-lactamase, sulfonamide resistant *sul*, and tetracycline-resistant ribosomal protection gene families were present exclusively in the modern sample (*SI Appendix,* Fig. S7, Dataset S7). When contrasting the St Leonard sample with the other ancient samples, and in particular the contemporaneous Irish sample, a higher occurrence of read matches with three gene families conferring resistance to macrolides, lincosamides and penicillin were found (ABC antibiotic efflux pump, non-erm 23S rRNA methyltransferase, and penicillin-binding protein) even though a larger sample may help in the future to assess the significance of this discrepancy (Dataset S8). In one individual (P45), matches with the gene *vanR* (glycopeptide resistance gene cluster) were also found. This gene was largely absent in the comparative dataset (i.e., 82% of the individuals) and was found at a higher occurrence only in one individual from the eighteenth–nineteenth century English data (ERR3003657).

## Discussion

Using optical polarised light microscopy, this study analysed calculus from 42 individuals buried in a late medieval cemetery associated with a leprosy hospital. This archaeological site is of national importance since excavations of leprosaria and/or their associated cemeteries are rare, not only in England. Through skeletal analysis, evidence of leprosy was detected in 66.7% (N = 28) of the skeletons sampled for this study (Dataset S1, *SI Appendix*). However, the absence of osteological evidence of leprosy in other skeletons sampled does not imply that they were not affected by the disease but rather that they may have died before the infection affected their skeleton^39,40^. When interpreting the data, it should be noted that all these individuals lived in the same buildings throughout the two phases of the hospital and potentially experienced similar diets and living conditions. Alternatively, Elma Brenner, in her analysis of monastic houses of Rouen, France, found that 'not all the resident lepers ate the same diet’ and this may be related to the religious status of the individual rather than to ideas about diet consumed as part of the care regime(^41^, pp. 96–97).

As seen in this study, the sample dimensions do not directly correlate with the number of findings identified. These data confirm how microremains are randomly entrapped in the calculus matrix and there are limitations in interpretation, including: (i) it is difficult to distinguish if the remains entered the mouth because of the food ingested or if they were inhaled, (ii) it is difficult to discern when the calculus formed during the individual’s life and consequently determine when a microremain was entrapped, (iii) it is impossible to establish the quantity of the original material that entered the mouth, and (iv) the absence of some types of microremains does not imply that they were not ingested or inhaled. With these limitations in mind, consulting research that documents diet and medical practices, and checking historical sources, may provide context for the presence of medical ingredients in calculus remains from medieval leprosaria.

St Leonard was supported by Peterborough Abbey and the people living in the hospital followed monastic rules^8,24^. This implies they were possibly following a diet ‘linked to preventative medicine and therapeutic treatments’ (^42^, p. 81). Their diet principally included cereals (from bread, soup, and ale), beans, vegetables, cheese and, to a lesser extent, fish, eggs, and meat. In both phases, evidence of cereals (Triticeae and Panicoideae), legumes (Fabaceae), and several fragments of vegetal remains (i.e., tissues, fibers, and vascular elements) that might suggest vegetable consumption were found. By contrast, the presence of an exotic spice (*Zingiber officinale*) was observed only in the individuals analysed from Phase 1. Except for ginger, no differences were found between males and females nor amongst individuals with leprosy or without skeletal evidence of the disease, suggesting a homogeneous regimen with potentially no differences between the sexes. However, people without leprosy evident in their skeletons may still represent people with the infection. The presence of the few starch grains associated with Panicoideae (e.g., millet) is challenging to justify since, although millet was known in late medieval Britain, it is rarely attested in the archaeobotanical record^43^. Future stable isotope analysis will clarify the meaning of these data.

The starch grains taxonomically assigned to ginger (*Zingiber officinale*) were found only in Phase I of the cemetery (eleventh–thirteenth centuries) and mainly in adult females (six of seven individuals). Six individuals (P9, P35, P45, P55, P69, P85) showed evidence of leprosy, and only one (P59) did not. Ginger was not identified in Phase II. It is possible that ginger was consumed but not preserved in the dental calculus of Phase II. The archaeological data shows that the frequency of leprosy was slightly higher in Phase I compared to Phase II^28^. This may suggest that more people were affected by this disease.

Moreover, looking at the spatial distribution of the burials^28^, three females consuming ginger were buried next to each other. This evidence may suggest that the three women were living in the same period at the hospital and potentially shared the same diet and medical care. The recommended use of ginger as a medicinal or culinary agent continued beyond the chronological range of Phase I. As the majority of the cases identified are female, it is also interesting that ginger in some instances is cited specifically for females. For example, Jong Kuk Nam notes that 'women with their teeth loosened by the cold can take wine in which ginger and galangal are boiled'(^28^, p. 333). As this is the first instance of ginger in the remains of a leprosarium, suitable comparative cases from other studies were difficult to find. Further research may yield more valuable data on the composition of dental calculus (and the presence of ginger and other spices) in medieval leprosaria.

There was no firm division between medicine and food in the past. As stated by Carole Rawcliffe in her analysis of leprosy in medieval England, 'the idea of distinguishing between diet and medication was as alien to the medieval practitioner as that of separating body and soul' (^8^, p. 214). Amending a patient's diet was an important first step in the treatment of disease in medical texts. For example, in her analysis of the account books and management of a fourteenth-century leprosarium in Barcelona, Spain, Clara Jáuregui examined the distribution of 'therapeutic food' and noted 'the first treatment was always a special diet,' including 'spiced water given as a therapeutic drink’ (^44^, p. 88). As stated previously, within the dietary protocols in the treatment of leprosy, Brenner found examples of differences in diets that depended on an individual's status. Generally, 'lepers were encouraged to consume foodstuffs that were mild and moist' according to the medical beliefs of the time (^41^, p. 95). The dual use of food and spices in the medieval period, as diet and medicine, is acknowledged when interpreting the data from the dental calculus analyses performed in this study.

According to Nam^28^, exotic ingredients, such as ginger, were 'essential medicines’ stored in monastic pharmacies. These species were imported via long-distance trade from Southeast Asia. Remedies were copied across different cultures, from classical learning to the influx of Arabic medicine in the medieval period, and they required ingredients which were not native to Northern Europe. While there were complaints, such as that of the mid-eighth-century Bishop Cyneheard of Winchester, who lamented that the remedies in his library were of little use since they require a number of ingredients of foreign origin (^45^, pp. 103–104), which suggests that access may have been restricted at times, supply came via the trade routes between Arabia and the East. Hunt records a cleansing syrup from an Anglo-Norman remedy book for people with scabies or leprosy; this was comprised of ginger and 18 other diverse ingredients, which Rawcliffe describes as 'an eclectic mixture of East and West’ (^8^, p. 216). Despite the frequency of occurrence in *materia medica* and written medical recipes, the long-distance origins of plant species, such as ginger, means that such ingredients must have been expensive and therefore not readily accessible to many people^46^.

In historical accounts, the term 'leprosy' does not always refer to the condition called leprosy or Hansen's disease today. In treating conditions considered to be 'leprosy' the remedies may not be directly targeted to the specific disease, but rather focused on affected body parts or major symptoms which may be shared by different conditions, such as general recipes for oral complaints, sore throats or vocal disruptions, headaches, skin eruptions such as scabies, wounds, inflammation, infections, swellings, or pain. In one contemporary text, Gilbertus Anglicus' *Compendium medicinae,* which was published in the early thirteenth century and later translated into Middle English versions, ginger appears in many recipes, including those for various afflictions of the tongue and teeth^47^. The recipes recommend rubbing or holding the medicine on the teeth, tongue, and gums. Other applications of ginger included medicines for the eyes, gargarismes (a gargle or mouthwash) for coughing and hoarseness or loss of voice, and as a means to purge the nose and head of corrupt humours (see *SI Appendix f*or citations). These are just a few examples of ginger in a contemporary medical text when considering the context surrounding the appearance of *Zingiber officinale* in dental calculus in the Phase I skeletons from a medieval English leprosarium. These recipes include body parts which can be severely affected by leprosy, such as the mouth, the eyes, the nose, and the vocal cords, and written instructions to hold and rub medicines against the teeth (where ginger fragments could have been encapsulated within a developing calculus matrix). In a record of an investigation of provisions allowed into the hospital of St Leonard, The White Book of Peterborough does not mention any spices or medicinal ingredients^48^. Jáuregui in her analysis of the account books of the leprosarium of Barcelona found a recipe for an infusion of raisins, a type of sugar (*sucre cordallat*), ginger, and liquorice for the treatment of an enslaved person in the hospital. Jáuregui currently is working on the first full transcription of the hospital's account books. It is unknown, at the time of this publication, if ginger occurs in other contexts apart from this recipe (see *SI Appendix f*or citation).

To our knowledge, this is the first study which has successfully identified *Zingiber officinale* in ancient human dental calculus in either England or elsewhere on the European continent. More importantly, it potentially represents the earliest archaeological evidence of the use of ginger as a medicinal ingredient. The significance of the recovery of *Zingiber officinale* from the human remains unearthed at St Leonard lies in the fact that, from a contextual point of view, it suggests that the leprosarium and its community used ginger as one of the treatments against the effects of leprosy. Specifically, these data show that ginger was found only in the calculus of seven individuals, mainly females, belonging to the first phase of the cemetery (eleventh–thirteenth centuries). By cross-referencing these data with the overall composition of the sample (13 females, 11 males and two individuals of undetermined sex), this study strongly suggests that women, in particular, may have been cared for using medicines that included ginger. Archaeological evidence from St Leonard shows that the presence of this exotic and expensive spice had a marked biological sex dimension. While it should be noted that ginger was used for symptoms of other diseases at this time, the fact that the people buried in this leprosy hospital cemetery had bone changes of leprosy strongly suggests that ginger was specifically administered to people with this infection. As it was an expensive compound it would have made sense to use it for a disease for which the hospital was founded.

Along with the finding of ginger, another key outcome of this research is the identification of insect hairs. Fragments of insect hairs, possibly dermestids, are rarely found in ancient calculus and some authors have considered this finding to be the results of post-excavation contamination, possibly due to pests in museum storage facilities^49^. In our study, they were found mainly embedded in the calculus matrix. No dermestid hairs were found in the laboratory dust traps nor in the analysed soil samples. In one case, they were mixed with several fragments of vegetal remains (i.e., tissues, fibers, and vascular elements*)* and fungal spores and found in the inner part of the calculus sample. If these findings are the result of *post-mortem* contamination, it is challenging to explain why they were found in the internal part of the calculus and mixed with microremains commonly not considered contaminants. If they were incorporated into the matrix during calculus development, they may be interpreted as non-dietary remains, present in the surrounding environment and incorporated via inhalation or ingestion.

### Leprosy and oral microbiomes

Metagenomic taxonomic analysis did not detect any authentic DNA molecular record of the microbial etiological agent of leprosy*, Mycobacterium leprae*, regardless of the documented presence of skeletal lesions consistent with leprosy (Dataset S1). This was observed by following an approach aimed at maximising sensitivity (the use of a database of complete genomes in Kraken2) and specificity (the use of multigene specific markers in MetaPhlAn3). However, multiple factors may potentially affect the presence of pathogen DNA in ancient calculus substrates. Primarily, the pattern of calculus deposition in relation to time and the severity of the infection on incorporation of bacterial DNA in calculus are important (e.g., the presence and position of leprosy-related oral lesions). Moreover, due to the low number of samples analysed in this study, more analyses are needed to unravel the preservation of *M. leprae* in the oral cavity and in particular in dental calculus.

Overall, our metagenomic survey indicated that leprosy did not significantly alter the oral microbiome of the individuals analysed when compared to a contemporaneous sample from medieval Ireland. This is in line with recent studies showing that dental calculus oral microbiomes do not preserve a record of health and disease^50^, whereas interindividual variation resulting from an individual's life history and ecology may be the major driver of oral microbial diversity in dental calculus.

While no taxonomic changes were detected, at the functional level the oral microbial community of the individuals from St Leonard was found to be enriched with some gene families that confer resistance to antibiotics (e.g., macrolides, beta-lactam, and penicillin). The presence of starch grains from ginger along with contemporary historical evidence suggests that people affected by leprosy may have been treated with medicinal herbs. The oral microbiome could be influenced by these treatments, a process that may have led to resistance to antimicrobial properties of medicinal herbs. The analysis of a larger number of samples in the future may help to ascertain to what degree functional changes in AMR associated with chronic long standing conditions like leprosy may be recorded in the dental calculus oral microbiota.

## Methods

The 2014 excavation of St Leonard leprosy hospital was undertaken by Archaeological Services, Durham University, England. The post-excavation report was published by the York Archaeological Trust in 2017 and is available online^24^. According to the archaeological report, the cemetery had two main phases of use. Phase I included 83 inhumations dating from the eleventh to the thirteenth centuries. Phase II, dating from the fourteenth to the sixteenth centuries, contained 47 inhumations. In the current study, data concerning biological sex, age at death, and the description of the skeletal evidence for leprosy were also collected and compared with the published anthropological report.

The sampling was conducted within the Fenwick Human Osteology Laboratory, Department of Archaeology, Durham University. Overall, sampling included 26 individuals from Phase I (11 males, 13 females, and two indeterminate individuals) and 16 individuals from Phase II (12 males and four females) (Dataset S1). Biological sex could not be estimated in two individuals because of poor skeletal preservation. The sampled individuals’ ages ranged from 18 to 45 years at death, but four individuals might be older than 45 (Dataset S1). In this study, a report was created to record the state of preservation of the dentition of each individual, the presence of oral pathologies, and the position of calculus on the teeth, its quantity, and its distribution through the dentition (Fig. 1b). The photographic documentation has been deposited in Zenodo (10.5281/zenodo.7643951). The files are not publicly accessible but are available upon request. To preserve calculus for future studies, individuals with very few patches of calculus were excluded from this research. More information about the sampling method is reported in the supplementary material. Since several analyses (e.g., chemical, microscopic, proteomic, aDNA) were performed, multiple samples were collected. This paper does not discuss the results of the chemical and proteomic analyses.

After sampling, the samples were transported to the DANTE-Diet and Ancient Technology Laboratory, Department of Oral and Maxillo Facial Sciences, Sapienza University of Rome to study the microremains through polarised light microscopy. Here, anti-contamination protocols were applied to prevent, or at least reduce, any possible laboratory contamination during the calculus decalcification procedures (see *SI Appendix)*. Next, the calculus was cleaned by removing soil deposits adhering to its surface using a method already established in previous research and improved during the MEDICAL project^13,51^ (Fig. 1c). Once the calculus was clean, it was dissolved in hydrochloric acid, and finally, the samples were prepared for polarised light microscopy. More information on the laboratory methods is available in the supplementary material.

Ancient DNA laboratory analyses were conducted in the dedicated aDNA facility of the Center of Molecular Anthropology for Ancient DNA Studies at the University of Rome “Tor Vergata”, following standard precautions for access to the facilities and decontamination^52^. From 6 to 10 mg of dental calculus from each tooth was sampled for DNA extraction with a silica-based method^53^. Double-stranded genomic libraries were constructed^54^ and sequenced in equimolar concentrations in paired-end mode (2 × 150 bp) in an Illumina HiSeqX platform (Macrogen Europe). The raw reads generated were adapter-trimmed, quality-filtered and submitted to taxonomic classification with Kraken2 and MetaPhlAn3. More information on the laboratory methods and the bioinformatic analysis are available in the supplementary material.

### Supplementary Information

Supplementary Information 1.

Supplementary Information 2.

## Data Availability

Data are available in the main text or the supplementary materials. The report on the excavation of St Leonard leprosy hospital, including the analysis of the skeletal remains analysed in this study, was published by York Archaeological Trust in 2017 (https://static1.squarespace.com/static/5c62d8bb809d8e27588adcc0/t/5ce6b8a3a4222f7ef96b9b8f/1558624461880/YAT-AY11-Midland-Road-Peterborough.pdf). The data from the dental calculus sampling have been deposited in Zenodo. The files are not publicly accessible but are available upon request (10.5281/zenodo.7643951). Codes used are available at https://github.com/claottoni/Peterborough-dental-calculus. All newly generated sequencing data have been deposited in the ENA repository (https://www.ebi.ac.uk/ena/browser/home ) under project accession PRJEB67363 (sequences accession numbers ERR12115113-ERR12115118).
