# Supplementary material for: First archaeological evidence for ginger consumption as a potential medicinal ingredient in a late medieval leprosarium at St Leonard, Peterborough, England
Source: Sci Rep. 2024 Jan 30;14:2452. doi: 10.1038/s41598-024-52422-8 (PMC10827753; doi:10.1038/s41598-024-52422-8)
Supplement: Supplementary file 1 — Supplementary Information 1. [file 41598_2024_52422_MOESM1_ESM.pdf]

Supporting Information for

First archaeological evidence for ginger consumption as a potential medicinal ingredient in a late medieval leprosarium at St Leonard, Peterborough, England

Elena Fiorin, Charlotte A. Roberts, Marica Baldoni, Erin Connelly, Christina Lee, Claudio Ottoni, Emanuela Cristiani

Corresponding author: Elena Fiorin, Email: elena.fiorin@uniroma1.it

This PDF file includes:

SI text
Figures S1 to S7
Tables S1 to S4
Legends for Dataset S1 to S8
SI References

Other supporting materials for this manuscript include the following:

Dataset S1 to S8

SI Text

Methods

Classification of bone changes for leprosy. In this study, skeletal lesions were tentatively classified into three diagnostic categories, describing skeletal changes that have been identified as specifically caused by leprosy and those that may be related to this disease (for a complete description of how leprosy can affect bones and teeth, a review of the skeletal changes for lepromatous leprosy, and all the bioarcheological studies conducted on skeletons from archaeological sites see Roberts (1) and references within): i) lepromatous leprosy: rhinomaxillary syndrome (RMS) alone, or RMS and hand or foot bone changes (or both hand and foot bone changes) and periosteal lesions on the lower leg bones; ii) possible leprosy within the context of a leprosarium cemetery: no RMS, but diagnostic bone changes of the hand and foot bones, and periosteal lesions on leg + foot and/or hand bones; iii) insufficient skeletal evidence for a diagnosis: no RMS or diagnostic bone changes of the hand and foot bones; only periosteal lesions on the lower leg bones present, bearing in mind there are many causes for this bone change.

Dental calculus sampling. The two main criteria that determined the calculus sampling protocol were whether teeth were preserved for an individual skeleton and the amount of calculus potentially available for analysis. The sampling procedure consisted of the removal of calculus from the tooth surface with a clean dental plastic scraper. The sample collected was immediately stored in a sterile plastic tube (1.5 mL). Plastic tubes were labelled with the individual's number, the tooth sampled, and the sampled calculus position. Permanent teeth were described following a system that avoided using lengthy tooth names. The letter 'U' indicated the upper dentition, and 'L' the lower dentition. Letters 'R' and 'L' were used to distinguish the right and the left sides. Teeth were identified using their initial letter (i.e., 'I' for the incisor) and a number to specify which teeth were referred to (i.e., 'I2' for the second incisor). Once the calculus samples were removed from the teeth, they were weighed and examined with a high-resolution stereo microscope with a zoom range of 16x (ZEISS Axio Zoom.v16). Using the stereo microscope, whether the calculus samples had a smooth and regular or highly porous and rough surface was noted.
In the sampled individuals, 19 (45.3%) showed clear signs of leprosy, 14 (33.3%) did not have sufficient skeletal evidence to suggest the person had leprosy, and nine (21.4%) were potentially affected by the disease (Dataset S1).

The anthropological data are in part modified from the original archaeological report (2) and, together with the calculus descriptions, are described in the supplementary information (Dataset S1). Regarding Phase I (eleventh- thirteenth centuries), 41 calculi samples were collected from 26 individuals. The sample weight ranged from 0.7 to 51.4 mg, and 261 slides were prepared for analysis. In the teeth sampled (N=41), 53.7% (N=22) were lower anterior teeth, 19.5% (N=8) were lower posterior teeth, 12.2% (N=5) were upper anterior teeth, and 14.6% (N=6) were upper posterior teeth. Calculus was mainly sampled from the buccal side of the teeth (51.2%, N=21), followed by the lingual (41.5%, N=17), the mesial (4.9%, N=2), and the mesial/distal sides (2.4%. N=1). From the 16 individuals of Phase II (fourteenth- sixteenth centuries), 25 calculus samples were collected. Their weights ranged from 0.8 to 79.3 mg, and 116 slides were studied. In the teeth sampled (N=23), 56.5% (N=13) were lower anterior teeth, 30.4% (N=7) were lower posterior teeth, 8.7% (N=2) were upper anterior teeth, and 4.4% (N=1) were upper posterior teeth. Calculus was mainly sampled from the buccal sides of the teeth (60%, N=15), followed by the distal (36%, N=9) and the lingual side (4%, N=1).

Anti-contamination protocols adopted. Anti-contamination protocols were applied to prevent, or at least reduce, any possible laboratory contamination during the calculus decalcification procedures (3). Instruments and working areas were systematically cleaned with 70% ethanol and boiling water. Powder-free gloves, masks, and polypropylene lab coats were worn, and most of the work was conducted under a laminar flow hood. During the decontamination procedures, only the person who was performing the analysis was allowed to enter the laboratory. Moreover, slide ‘traps’ were placed around the laboratory to monitor airborne contaminants. The contaminants recovered from the traps were excluded from the study (Figure S4).

Calculus cleaning and decalcification protocol. The soil was manually removed under a stereomicroscope at a magnification of up to 100X. Plastic tweezers were employed to hold the sample, and with a sterile needle soaked with drops of 0.5 or 1.5 M hydrochloric acid, the soil attached to the exterior stratum of the plaque was gradually dissolved and scraped off. Milli-Q water was used to wash and remove potential contaminants. Regarding the very small samples, a fine paintbrush with nylon bristles, modified for this specific step, was employed instead of the needle. Once the calculus surface was cleaned and all the residues of the external layer were removed, the samples were washed in a new plastic tube filled with Milli-Q water and placed in a minicentrifuge up to three times to remove any traces of loose sediment. The samples were then dried and stored in the same plastic tube. The most challenging samples were inspected again with the ZEISS Axio Zoom.v16 to check if there were any traces of impurities. In cases where some contaminated areas remained, the samples went through the decontamination procedures again. Next, the microremains were extracted. Depending on the quantity of the calculus deposit, 0.5 - 1 mL of 0.5 M - 1.5 M hydrochloric acid was added to the plastic tube to decalcify the samples. With the larger samples, an ultrasonic cleaner bath for at least 5 minutes was used (Argo Lab ultrasonic cleaner bath, AU-32) to accelerate the decalcification process. Once decalcification was complete, the samples were mounted on glass slides. Generally, 2 drops of the sample and 2 drops of a solution of 50:50 glycerol and Milli-Q water were added onto a glass slide with a 22x40 mm cover glass. The slides were then observed under brightfield and cross-polarised light on a motorised Zeiss Axio Imager M2 with magnifications up to 1000x and coupled with an Axiocam 305 colour camera. Microremains were photographed, measured, described, and then compared with a modern reference collection of plants native to Europe, including the Mediterranean, stored at DANTE Laboratory and with descriptions from published literature. In addition, as an objective of the MEDICAL project, a modern reference collection of medicinal plants mentioned by medieval authors in relation to the treatment of leprosy was created. The plant specimens were sampled at the Botanical Garden of Rome, which is part of the Department of Environmental Biology of the Sapienza University of Rome or bought at herbal shops.

Ancient DNA extraction and library preparation. Laboratory analyses were performed in the dedicated ancient DNA (aDNA) facility of the Center of Molecular Anthropology for Ancient DNA Studies of the University of Rome “Tor Vergata”. The Centre features state-of-the-art laboratories for aDNA consisting of individual ‘cleanroom quality’ working spaces (ancient sample pre-

treatment, milling, DNA extraction, genomic library and PCR set-up fully equipped rooms). Modern DNA and post-PCR laboratories are available in separate buildings at the Department of Biology.
For each individual between 6.5 and 10.4 mg of dental calculus from one single tooth were sampled for DNA extraction following silica-based methods (4). Calculus flakes were gently broken by a sterile spatula and washed in 1 mL of EDTA 0.5M pH 8.0 for 15 min. in orbital rotation at 50°C. After decanting the samples by centrifuge, the pellet was incubated in 1 mL of lysis buffer (EDTA 0.5 M pH 8.0 and 0.25 mg/mL proteinase K) for 12 h at 55°C and a subsequent 36 h at 37°C in a thermoshaker (500 rpm). Samples were decanted (3 min. 13000 rpm) and purified by using the Roche Assembly Tubes silica columns using an in-house binding buffer (5M Guanidine hydrochloride MW 95.53, 40% Isopropanol, 90 mM Sodium Acetate, 0.05% Tween-20 10%) (5). Samples were eluted twice in 50 µl of TET buffer (10 mM Tris-HCl pH 8.0, 1 mM EDTA, 0.05% Tween-20 10%) and DNA extracts were stored in freezer at -20°C. Two negative controls were added in the extraction batch. Residual pellets were also stored in the freezer at -20°C for potential further analyses.
For each sample 20 µL of DNA extract were converted in double-stranded genomic libraries as described by Meyer and Kircher (6) and Kircher et al (7), together with a library negative control. A total of 15 µL of each library were split in three reactions and used as a template in 50 µL volume for double-indexing PCR (25 cycles; PfuTurbo Cx Hotstart polymerase 0.05 U/µL, Pfu Turbo Cx reaction buffer 1X, dNTPs 200 µM each, and primers P5 and P7 200 nM each). Negative controls were added for each amplification reaction. A high number of amplification cycles were chosen in order to amplify the libraries of the negative controls and sequence them. The triplicate amplified products were then pooled and purified with the Minelute purification kit (Qiagen). Reconditioning PCR was performed for removing the heteroduplex due to over-amplification during indexing PCR. A total of 2µL of purified amplified libraries were added in 21 µL AccuPrime Pfx Supermix and primers IS5 and IS6 (200 nM each) for 1 cycle reaction. The reconditioned products were finally purified with the MinElute purification kit (Qiagen). The quality check of the libraries was performed in an Agilent Bioanalyzer 2100 (High Sensitivity DNA kit). Quantification was then performed by the fluorometer Qubit4 using the double stranded High Sensitivity DNA kit. The libraries were sequenced in equimolar concentrations in paired-end mode (2 x 150 bp) in an Illumina HiSeqX platform (Macrogen Europe).

Quality filtering of the raw reads and taxonomic classification. Supercomputing infrastructure for high-throughput sequence data analysis is provided by the computational infrastructure of Cineca, one of the Large-Scale Facilities in Europe. Codes used are available at https://github.com/claottoni/Peterborough-dental-calculus. The raw reads were checked for quality by FastQC (8) and the adapters trimmed with AdapterRemoval (--minlength 30 --minquality 25 --collapse) (9). The taxonomic classification of the merged trimmed reads was done with Kraken2 (10) using a 122 Gb custom database built in February 2022 and containing complete genome sequences of Bacteria, Archaea, Viruses, and Fungi from NCBI Refseq and GenBank. Species abundance was estimated with Bracken (-r 65 –t 50) (11). To test whether the reads classified by Kraken2 are distributed throughout a reference genome, in particular for *Mycobacterium leprae*, or originate from small fragments of it (hence most likely false positives) a second run was done with the additional flag --report-minimizer-data. Genome length normalisation of read abundances was performed using a custom script previously used (12) and available on GitHub (https://github.com/claottoni/toolbox). To test the presence of *Mycobacterium leprae* in the dental calculus samples investigated with a more specific multigenic metagenomic approach, the taxonomic classification was also conducted with MetaPhlAn3 (13).
We tested whether DNA originating from plants (Viridiplantae), in particular from the genus *Zingiber*, was present in the dental calculus samples analyzed by interrogating a Kraken2 custom database of plastid and mitochondrial genomes from the NCBI. No reads belonging to the genus *Zingiber* were found. We acknowledge that analyses of plant and animal DNA from shotgun metagenomic datasets are often problematic due to spurious hits causing false positive signals and as a result of limitations and assumptions associated with the use of modern reference sequences that may not reflect ancient taxa. For this reason, samples were not further screened for plant (and animal) DNA.

Authentication of the metagenomics data. To evaluate potential contamination from other microbial sources, the source composition of the analysed calculi was predicted by SourceTracker (14) using the sourcetracker_for_qiime.r script (https://github.com/danknights/sourcetracker). Furthermore, the reads were aligned to the reference genomes of the most abundant species in the analysed samples using bwa aln (15); (-n 0.1). The presence of post-mortem damage associated with cytosine deamination was assessed with mapDamage (16). To evaluate the edit-distance distribution, bed files were generated by bedtools bamtobed (-tag NM) (17) and the parameter −Δ% (negative difference proportion) (18) was calculated in R. Further estimation of the edit distance and post-mortem damage was also done after filtering the bam files for mapping quality and damage level with pmdtools (--requiremapq=30 --treshold 1) (19). Taxonomic classification was considered reliable for −Δ% values higher than 0.8.

Downstream analysis. Full taxonomic ranks for every species ID in the abundance tables were retrieved with Taxaranks (20) and pasted to the abundance tables with a custom script (https://github.com/claottoni/toolbox). The abundance tables were then filtered for Bacteria and Archaea and imported in R.
Microbial abundances were analysed with the package Phyloseq (21) in R. Taxa abundance, full taxonomy, and metadata were all stored in a biom object. Species abundances were normalised for library size through total sum scaling and then filtered to include only taxa present at >0.02%. To account for the compositional nature of microbiome data, a centered-log ratio (CLR) transformation with the package microbiome in R was applied. A non-metric multidimensional scaling (nMDS) of Aitchison distances calculated from the species abundances of different microbiome sources from this research and from the literature was done with Phyloseq and the *ordinate* function (22). Differences in the microbial composition among the dental calculus oral microbiomes investigated were tested on Euclidean distances of CLR-transformed species abundances by PERMAVOVA with Adonis test (999 permutations, significance threshold was set to p=0.01). For each comparison the difference in samples' variance was tested with betadisper and anova in R. Differentially abundant species in the groups of dental calculus microbiomes analysed were found with DESeq2 in R (23).

To conduct the AMR investigation extract_kraken_reads.py from KrakenTools, was used to extract and convert to fasta the reads of the ancient and modern calculus samples from this study and the literature classified as bacteria in Kraken2 (using the options -t 2 --include-children). The extracted reads were aligned against the CARD database with blastn (24). Then, a strategy based on the top bitscore match was adopted to retrieve the ARO accession number and the AMR gene family, as previously described (12, 25). To account for potential taphonomic conditions leading to preservation biases across samples of different chronology, blastn was used to align the reads against the nucleotide sequences of three microbial housekeeping genes (recA, rpoB, gyrB) deposited in the NCBI. The sequences were downloaded with *esearch* and *efetch* from EDirect package of NCBI. For each calculus sample, the number of reads matching the AMR gene families was normalized for the number of reads matching the three housekeeping genes and converted to counts per million. A total of 33 gene families were identified and the abundances were analysed to generate boxplots in the chronological samples with ggplot in R (Dataset S7, Figure S7). Pairwise Wilcoxon Rank Sum Tests were done in R with dplyr and the Benjamini & Hochberg p-value adjustment method (Dataset S8).

Results

Starch grain morphotypes observed in the dental calculus

Type I was observed in 14 individuals (N=> 59) (Dataset S2, Figure S1a, S1b, and S1c). Some of the starch grains were damaged (Figures S1g and S1p). Size, morphology, and a bimodal distribution characterising grains of this type are encountered in members of the plant tribe Triticeae (Poaceae family) (26, 27). The large grains (A-Type) have a 2D oval/circular shape and

a 3D lenticular shape, while small grains (B-Type) have a 2D oval/circular shape and a 3D spherical/ovoidal shape. The hilum is centric and closed; the extinction cross is radially symmetrical. In the large grains, lamellae are visible, concentric, and distinct. The diameter/central axis of A-type starch grains ranges between 43 and 17 μm (mean size of 28 μm) in Phase I and between 61 and 18 μm (mean length of 35 μm) in Phase II. B-type grains, few in number and usually <10 μm, are rarely diagnostic to taxa and were not considered (28).

Type II was observed in 10 individuals (N=22) (Dataset S2, Figures S1h and S1i). These grains have a polyhedral to sub-polyhedral morphology, with occasional oval morphology. The hilum is centric with fissures; sometimes, the hilum is open. The extinction cross is radially symmetrical. The grain size ranges between 13 and 27 μm (mean size of 20 μm) in Phase I and between 18 and 26 μm (mean length of 21 μm) in Phase II. These features are consistent with starch grains of the Panicoideae (Poaceae family) (29–32). Santiago-Marrero et al. (33) divided these types of starch grains into two classes based on the dimensions of the granules. They associated starch grains with a grain size range of 25.58–15.15 μm with an average of 19.2548 μm for the large Panicoideae taxa (see also 30, 34, 35).

Type III was detected in four individuals (N=4) (Dataset S2, Figures S1k, S1l and S1m). The grains have a 2D ovate/oval shape with a protrusion or reniform shape with an elongated hilum, very visible lamellae, and sometimes a depressed hilum. In two granules, a deep longitudinal fissure is present. The extinction cross is bilaterally symmetrical. The main axis ranges between 29 and 38 μm (mean size of 35 μm) for both phases. They can be attributed to Fabaceae (35–38).

Type IV was observed in seven individuals (N=15) (Dataset S2). An ovoid-elongated bell shape characterises these grains; no fissures or lamellae were observed. Under cross-polarised light, an eccentric hilum is typically visible toward the narrow end. The extinction cross usually has two arms with a wavy morphology (the arm looks like a brace). The main axis (maximum length) ranges between 19 and 53 μm (mean size of 33 μm), and the full-width ranges between 18 and 37 μm (mean size of 28 μm). According to the literature and our reference collection, these features are consistent with starch grains of *Zingiber officinale* of the Zingiberaceae family (38–40). Due to some morphological features (hilum, shape, and lamellae), we excluded those belonging to the bulbs of *Lilium* sp. (40–43) or other tubers that were grown in medieval England, such as turnip, parsnip, and horseradish (44).
Regarding the measures, the maximum length of the *Zingiber officinale* modern starch grains is, on average, less than *Curcuma longa* and *Alpinia officinarum.* Conversely, the maximum width of the three species is highly variable (Figure S3). However, on average, *Alpinia officinarum* starch grains are narrower, and those of *Curcuma longa* are wider when compared with the *Zingiber officinale* starch grains. In summary, comparing the measurements and the morphological features of the three modern species, the archaeologically preserved starches found at St Leonard are similar to those of *Zingiber officinale*.

Type V starch grains were observed in two individuals (N=> 51) (Dataset S2). They have an oval and/or triangular shape. Lamellae were rarely visible, and a central depression characterised some of them. The hilum is mainly centric, and the extinction cross has bent arms. The cluster found in an adult female (P59) contained starch grains with different lengths (between 2 and 23 μm). These features could be compatible with those observed in starch grains associated with Fagaceae (33, 45, 46).

Historical manuscript references

Clara Jáuregui, personal communication 16 May 2023; ACB (Arxiu Capitular de Barcelona), Hospitals, Libre de comptes dels mesells, 1379-1380, fol. 145r: "Item, panses, sucre cordallat, gingebre, regalicia e altres cosas per fer aygua ha ops del sclau".

Anglicus, Gilbertus. 1510. *Compendium Medicinae Gilberti Anglici: tam morborum universalium quamparticularium nondum medicis sed cyrurgicis utilissimum*, Lyon: Vincent de Portonariis (Google Books);
https://www.google.com/books/edition/Compendium_medicine_Gilberti_Anglici_tam/n9VFmZG7ew4C?hl=en&gbpv=0

- teeth, tongue, and gums: e.g., pp. 334, 343
- medicines for the eyes: pp. 278, 283, 297
- coughing and hoarseness or loss of voice: pp. 379, 390
- tightness of the chest (with coughing and hoarseness): p. 381
- hot and moist diet (dieta calida et humida) to treat coughing: p. 389
- purge the head of humours: p. 213

Page numbers refer to those assigned in the downloadable pdf of the edition available from Google Books at the link provided. The authors are aware of the different approaches to numbering or foliating early printed editions. Please contact the authors with any questions. The authors also consulted Faye M. Getz's edition of a later Middle English translation of the text: *Healing and Society in Medieval England: A Middle English Translation of the Pharmaceutical Writings of Gilbertus Anglicus*, University of Wisconsin Press, 1991. *ProQuest Ebook Central*, https://ebookcentral.proquest.com/lib/warw/detail.action?docID=3445339 .

SI figures

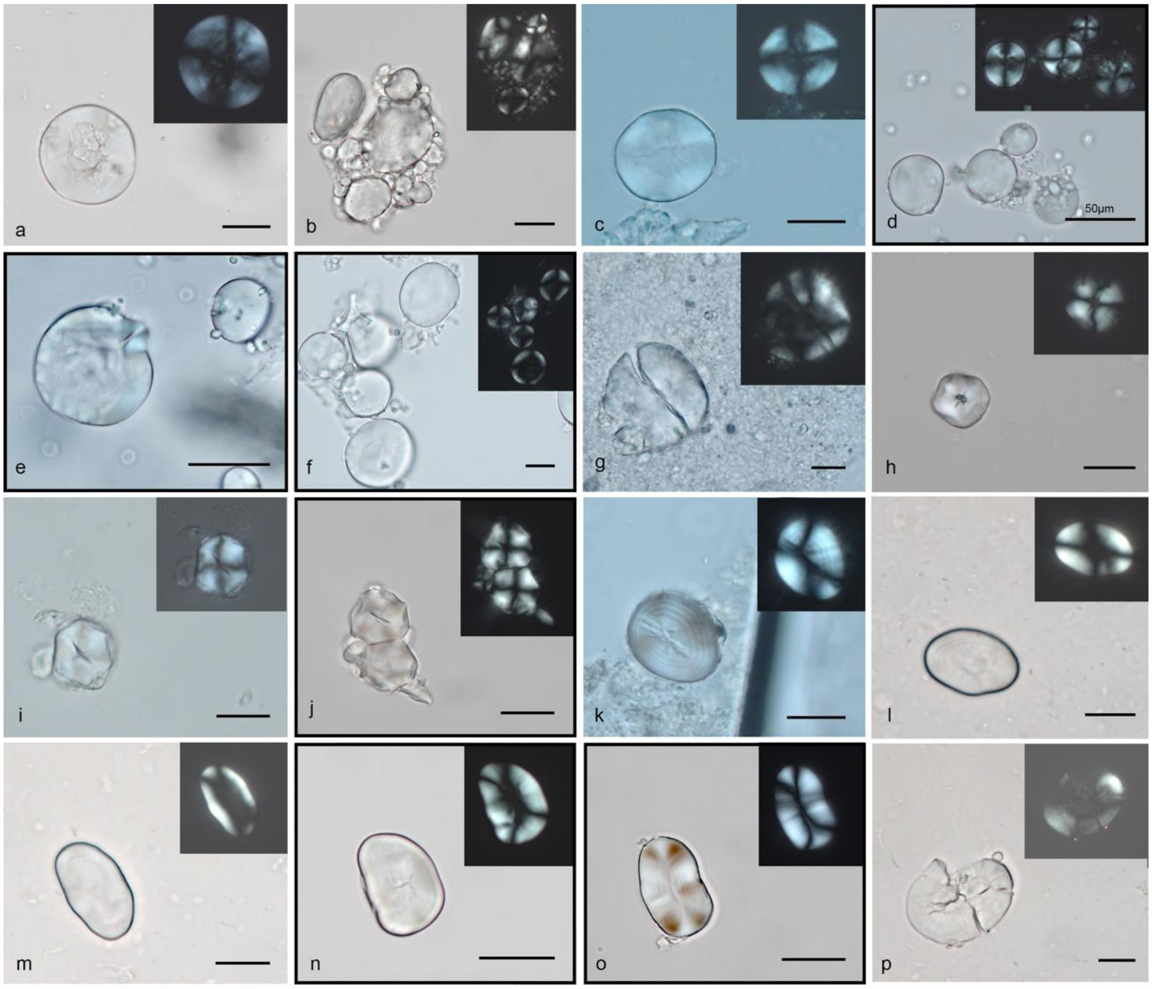

Figure S1. Archaeological starch grains identified in the calculus of St Leonard individuals and experimental reference (black-framed photos). Starch grains are shown in bright field and cross-polarised light. Scale bars, when not specified, are 20 µm. (**a**) Type **I** (P17); (**b**) Type **I** (P112); (**c**) Type **I** (P120); (**d**) *Triticum aestivum*; (**e**) *Secale cereale*; (**f**) *Hordeum vulgare*; (**g**) damaged starch grains (P91). Due to the presence of A and B-Type granules, the starch grains belong to the plant tribe *Triticeae* (Type **I**); **(h**) Type **II** (P4); (i) Type **II** (P16); (**j**) *Sorghum bicolor*; (**k**) Type **III** (P42); (**l**) Type **III** (P82); (**m**) Type **III** (P 96); (**n**) *Pisum sativum*; (**o**) *Lens culinaris*; (**p**) damaged starch grain (P96).

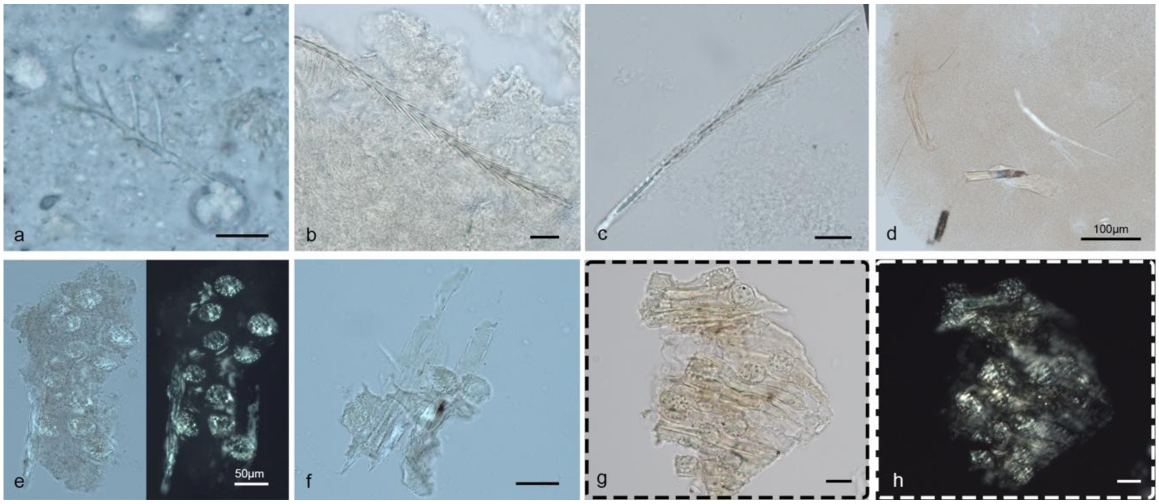

Figure S2. Archaeological microremains from dental calculus of the St Leonard individuals and experimental reference (black and white dotted framed photos). Some microremains are shown in bright field and cross-polarised light. Scale bars, when not specified, are 20 µm. (**a**) Possible bee hair (P84); (**b,c**) Insect hair (dermestid?) (P120, P83); (**d**) Insect fragments, vegetal elements and fungal spores embedded in the calculus matrix (P83); (**e,f**) Vegetal elements entrapped in the calculus matrix (P42); (**g,h**) Vegetal elements found in *Lolium arundinaceum* spikelet, like those found in P42.

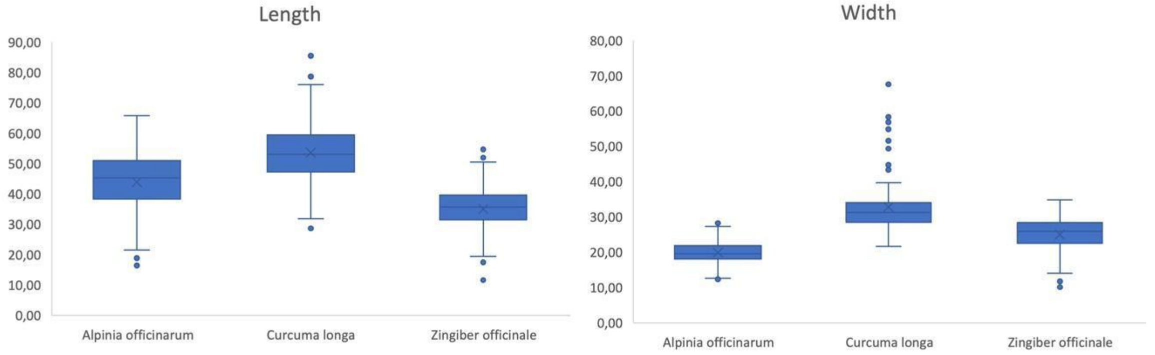

Figure S3. The distribution of starch grain length and width in *Alpinia officinarum*, *Curcuma longa*, and *Zingiber officinale* species (Zingiberaceae).

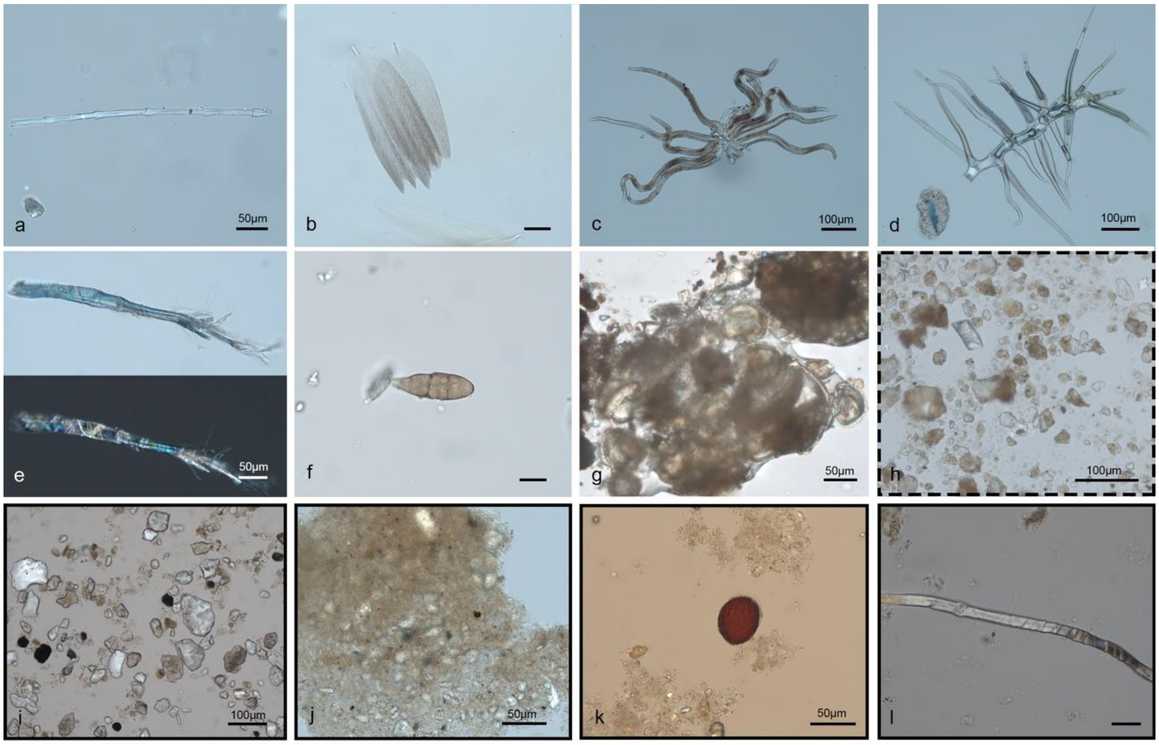

Figure S4. Contaminants were sampled from the laboratory dust traps, a soil sample from the burial (black-dotted framed photo), and contaminants observed in the external layer of dental calculus (black-framed photos). Scale bars, when not specified, are 20 µm. (**a**) Barbule fragment; (**b**) Lepidoptera wing scales; (**c,d**) Trichomes; (**e**) Vegetal fibre; (**f**) Fungal spore; (**g)** Pollen grains and dust; **(h**) Burial soil from St Leonard, Peterborough; (**I,j**) Residues of soil (P23, P83); (**k**) Non-pollen palynomorph (P42); (**l**) Vegetal fibre (P9).

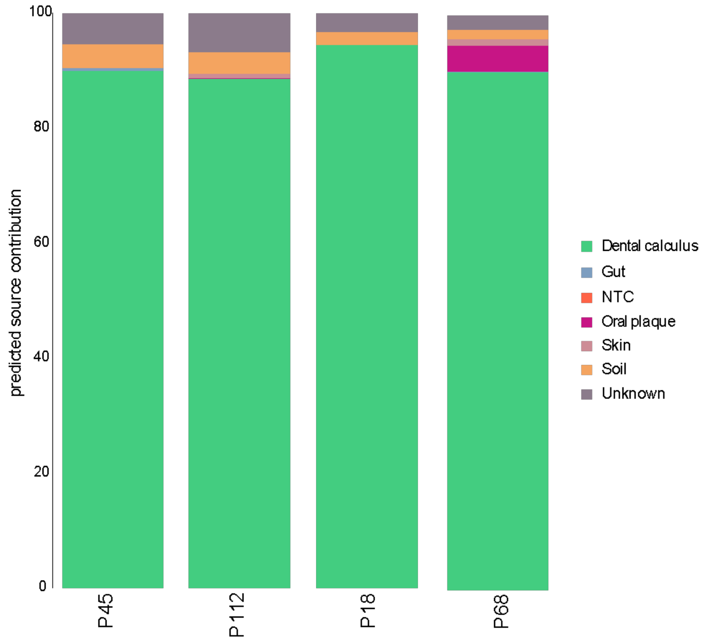

Figure S5. Stacked barplot of Sourcetracker analysis providing the predicted source contribution of Kraken2 classified reads. The proportion of reads originating from dental calculus and other microbiome sources is reported in Table S2.

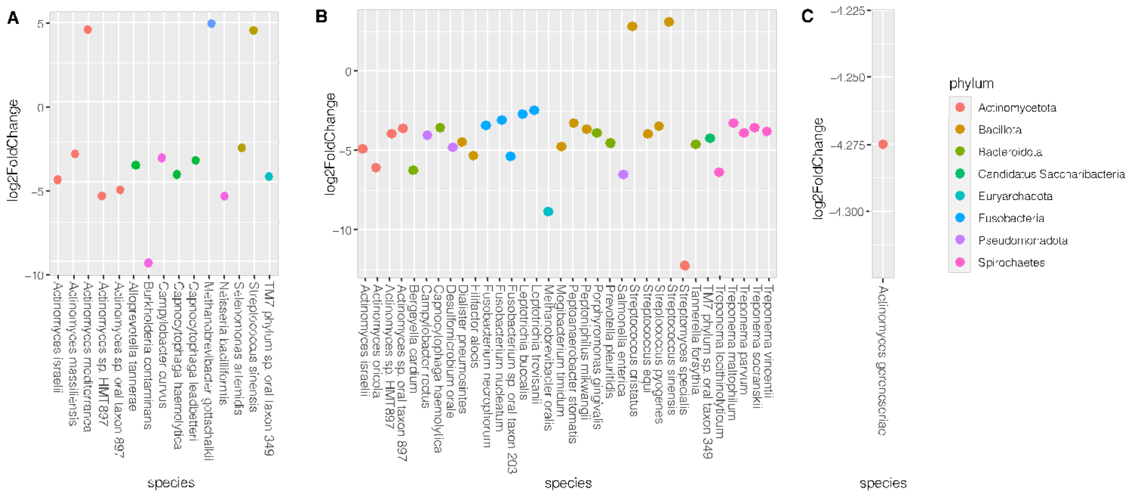

Figure S6. Differentially abundant species (Bacteria and Archaea) detected with DESeq2 between St Leonard and modern calculus samples (A), 18th-19th centuries CE UK (B), and medieval Ireland (C) dental calculus. Only the most abundant (baseMean>1000) and significant (padj<0.1) taxa are reported. Species significantly more abundant at Peterborough possess a positive log2FoldChange. The phylum of each identified species is color coded as in the legend.

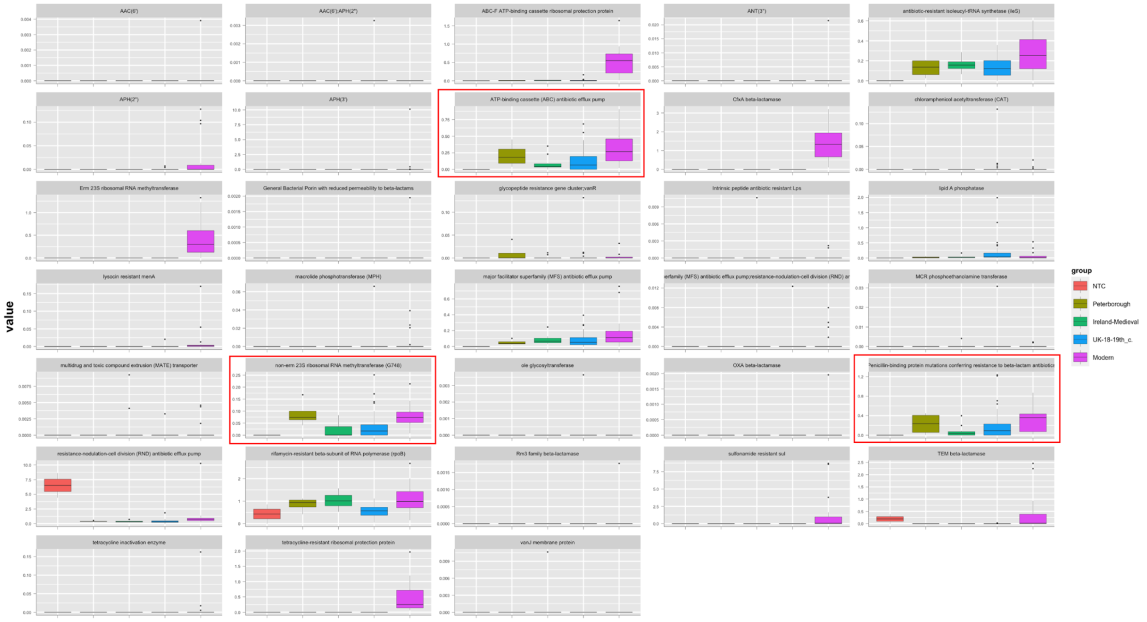

Figure S7. Boxplots representing the abundance of reads matching antibiotic microbial resistance (AMR) gene families. Samples from St Leonard analysed in the present research were contrasted with other medieval, historic (18th-19th centuries CE), and modern calculus samples from the published literature along with laboratory negative controls (NTC). Gene families found to be more abundant in the St Leonard individuals than in the contemporary sample from medieval Ireland are highlighted in red.

Table S1. Total reads generated after sequencing and quality filtering and total reads classified with Kraken2.

Sample ID	calculus (mg)	Total paired reads	Merged filtered reads	% Bacteria	% Archaea
P45	8.2	22,436,167	20,956,405	52.31	0.13
P68	8.3	16,981,011	16,717,240	45.65	0.04
P18	6.5	12,849,863	12,701,614	43.03	0.07
P112	10.4	21,217,027	20,899,262	32.80	0.25

Table S2. Results of Sourcetracker analysis reporting the estimation of the percentage of reads stemming from different microbiome sources.

Sample_ID	calculus	gut	NTC	plaque	skin	soil	unknown
P45	90.0%	0.5%	0.0%	0.0%	0.0%	4.2%	5.3%
P112	88.6%	0.1%	0.0%	0.1%	0.8%	3.7%	6.8%
P18	94.5%	0.0%	0.0%	0.0%	0.0%	2.2%	3.3%
P68	90.1%	0.0%	0.0%	4.6%	1.1%	1.6%	2.5%

Table S3. Results of PERMANOVA analysis - Adonis test (999 permutations) performed on CLR-transformed Euclidean distances calculated from microbial species abundances in the chronological groups of this study. The sample from St Leonard (PB) was compared with the medieval sample from Ireland (IRE), the 18th-19th century sample from England (UK), and the modern sample (MOD). The asterisk indicates significance below 0.01 threshold. The results of the dispersion test for variance homogeneity are reported in Table S4.

	Df	SumsOfSqs	MeanSqs	F.Model	R2	Pr(>F)
**PB *vs* IRE**						
Group1	1	1921	1921	1.498	0.091	0.156
Residuals	15	19234	1282	NA	0.909	NA
Total	16	21154	NA	NA	1	NA
**PB *vs* UK**						
Group1	1	2108	2108	1.155	0.023	0.294
Residuals	50	91287	1826	NA	0.977	NA
Total	51	93395	NA	NA	1	NA
**PB *vs* MOD**						
Group1	1	2142	2142	2.985	0.130	0.006*
Residuals	20	14350	718	NA	0.870	NA
Total	21	16492	NA	NA	1	NA

Table S4. Results of the dispersion test associated with the PERMANOVA analysis reported in Table S3. from microbial species abundances in the different chronological groups of this study. The sample from St Leonard (PB) was compared with the medieval sample from Ireland (IRE), the 18th-19th century sample from England (UK), and the modern sample (MOD). Significance threshold was set at 0.01.

	Df	Sum Sq	Mean Sq	F value	Pr(>F)
**PB *vs* IRE**					
Groups	1	192	192	1.171	0.296
Residuals	15	2454	164	NA	NA
**PB *vs* UK**					
Groups	1	778	778.	2.918	0.093
Residuals	50	13333	267	NA	NA
**PB *vs* MOD**					
Groups	1	2.60	2.60	0.057	0.813
Residuals	20	910	46	NA	NA

Dataset (separate file). The Dataset consists of an Excel file with eight separate sheets.

The first sheet (**Dataset S1**) contains the list of the dental calculus samples recovered from the individuals buried at St Leonard leprosy hospital. For each sample, the individual laboratory code, the biological sex, the age at death, the skeletal evidence of leprosy, the tooth sampled and its weight, the number of microscope slides observed, and the results of the polarised optical microscopy analysis are described. In addition, there is a brief description of the samples employed for aDNA analysis. The second sheet (**Dataset S2**) contains the types of starch grain found in the individuals analysed in this study. The third sheet **(Dataset S3)** contains the species abundance table after normalization for sequencing depth and genome length. The fourth sheet **(Dataset S4)** reports the statistics of alignment of the raw reads to the reference genomes of some of the most represented species detected by Kraken2. The fifth sheet **(Dataset S5)** contains the abundance data from MetaPhlAn3 analysis. **Dataset S6** reports the metadata associated with the samples used for the metagenomic analysis in this study and in the published literature. **Dataset S7** shows the abundance of reads mapping the AMR gene families in the Comprehensive Antibiotic Resistance Database (CARD). **Dataset S8** shows the P-values (Benjamini & Hochberg adjusted) of Pairwise Wilcoxon Rank Sum Tests in AMR genes family abundance (normalized for the number matching three housekeeping genes) investigated in the dental calculus samples from this study and the published literature.

SI References

1. Roberts, C. A. *Leprosy: Past and Present* (University Press of Florida, 2020).

2. Mc Comish, J. M., Millward, G., Boyle, A. “The Medieval cemetery of St Leonard’s leper hospital at Midland Road” (YORK ARCHAEOLOGICAL TRUST, 2017).

3. Crowther, A. Haslam, M., Oakden, N., Walde, D., Mercader, J. Documenting contamination in ancient starch laboratories. *J. Archaeol. Sci.* **49**, 90–104 (2014).

4. Dabney, J. *et al.*, Complete mitochondrial genome sequence of a Middle Pleistocene cave bear reconstructed from ultrashort DNA fragments. *Proc. Natl. Acad. Sci. U. S. A.* **110**, 15758–15763 (2013).

5. Dabney, J., Meyer, M. “Extraction of Highly Degraded DNA from Ancient Bones and Teeth” in *Ancient DNA: Methods and Protocols*, Shapiro B, Barlow A, Heintzman PD, Hofreiter M, Paijmans JLA, Soares AER, Ed. (New York: Springer, 2019), pp. 25–29.

6. Meyer, M., Kircher, M. Illumina sequencing library preparation for highly multiplexed target capture and sequencing. *Cold Spring Harb. Protoc.*, db.prot5448 (2010).

7. Kircher, M., Sawyer, S., Meyer, M. Double indexing overcomes inaccuracies in multiplex sequencing on the Illumina platform. *Nucleic Acids Res.* **40**, e3 (2012).

8. Andrews, S. *et al.*, FastQC: a quality control tool for high throughput sequence data (2010).

9. Lindgreen, S. AdapterRemoval: easy cleaning of next-generation sequencing reads. *BMC Res. Notes* **5**, 337 (2012).

10. Wood, D. E., Lu, J., Langmead, B. Improved metagenomic analysis with Kraken 2. *Genome Biol.* **20**, 257 (2019).

11. Lu, J., Breitwieser, F. P., Thielen, P., Salzberg, S. L. Bracken: estimating species abundance in metagenomics data. *PeerJ Comput. Sci.* **3**, e104 (2017).

12. Ottoni, C. *et al.*, Tracking the transition to agriculture in Southern Europe through ancient DNA analysis of dental calculus. *Proc. Natl. Acad. Sci. U. S. A.* **118** (2021).

13. Beghini, F. *et al.*, Integrating taxonomic, functional, and strain-level profiling of diverse microbial communities with bioBakery 3. *Elife* **10**, e65088 (2021).

14. Knights, D. *et al.*, Bayesian community-wide culture-independent microbial source tracking. *Nat. Methods* **8**, 761–763 (2011).

15. Li, H., Durbin, R. Fast and accurate short read alignment with Burrows–Wheeler transform. *Bioinformatics* **25**, 1754–1760 (2009).

16. Ginolhac, A., Rasmussen, M., Gilbert, M. T. P., Willerslev, E., Orlando, L. mapDamage: testing for damage patterns in ancient DNA sequences. *Bioinformatics* **27**, 2153–2155 (2011).

17. Quinlan, A. R., Hall, I. M. BEDTools: a flexible suite of utilities for comparing genomic features. *Bioinformatics* **26**, 841–842 (2010).

18. Hubler, R. *et al.*, HOPS: automated detection and authentication of pathogen DNA in archaeological remains. *Genome Biol.* **20**, 280 (2019).

19. Skoglund, P. *et al.*, Separating endogenous ancient DNA from modern day contamination in a Siberian Neandertal. *Proc. Natl. Acad. Sci. U. S. A.* **111**, 2229–2234 (2014).

20. Meng, G., Li, Y., Yang, C., Liu, S. MitoZ: a toolkit for animal mitochondrial genome assembly, annotation and visualization. *Nucleic Acids Res.* **47**, e63–e63 (2019).

21. McMurdie, P. J., Holmes, S. phyloseq: an R package for reproducible interactive analysis and graphics of microbiome census data. *PLoS One* **8**, e61217 (2013).

22. Oksanen, J., Guillaume Blanchet, F., Kindt ,R., Legendre, P., Minchin, P. R., O’hara, R. B., Simpson, G. L., Solymos, P., Stevens, M. H. H., Wagner, H. Vegan: Community Ecology Package. R package version 2.5-7 (2020).

23. Love, M. I., Huber, W., Anders, S. Moderated estimation of fold change and dispersion for RNA-seq data with DESeq2. *Genome Biol.* **15**, 550 (2014).

24. Jia, B. *et al.*, CARD 2017: expansion and model-centric curation of the comprehensive antibiotic resistance database. *Nucleic Acids Res.* **45**, D566–D573 (2017).

25. Brealey, J. C. *et al.*, Dental calculus as a tool to study the evolution of the mammalian oral microbiome. *Mol. Biol. Evol.* (2020) https:/doi.org/10.1093/molbev/msaa135.

26. Stoddard, F. L. Survey of Starch Particle-Size Distribution in Wheat and Related Species. *Cereal Chemistry Journal* **76**, 145–149 (1999).

27. Henry, A. G., Piperno, D. R. Using plant microfossils from dental calculus to recover human diet: a case study from Tell al-Raqā'i, Syria. *J. Archaeol. Sci.* **35**, 1943–1950 (2008).

28. Yang, X., Perry, L. Identification of ancient starch grains from the tribe Triticeae in the North China Plain. *J. Archaeol. Sci.* **40**, 3170–3177 (2013).

29. D'Agostino, A. *et al.*, Neolithic dental calculi provide evidence for environmental proxies and consumption of wild edible fruits and herbs in central Apennines. *Commun Biol* **5**, 1384 (2022).

30. Madella, M., Lancelotti, C., García-Granero, J. J. Millet microremains—an alternative approach to understand cultivation and use of critical crops in Prehistory. *Archaeol. Anthropol. Sci.* **8**, 17–28 (2016).

31. Lucarini, G., Radini, A., Barton, H., Barker, G. The exploitation of wild plants in Neolithic North Africa. Use-wear and residue analysis on non-knapped stone tools from the Haua Fteah cave, Cyrenaica, Libya. *Quat. Int.* **410**, 77–92 (2016).

32. Lippi, M. M., Foggi, B., Aranguren, B., Ronchitelli, A., Revedin, A. Multistep food plant processing at Grotta Paglicci (Southern Italy) around 32,600 cal BP. *Proceedings of the national Academy of Sciences* **112**, 12075–12080 (2015).

33. Santiago-Marrero, C. G., Tsoraki, C., Lancelotti, C., Madella, M. A microbotanical and microwear perspective to plant processing activities and foodways at Neolithic Çatalhöyük. *PLoS One* **16**, e0252312 (2021).

34. Liu, L. *et al.*, Fermented beverage and food storage in 13,000 y-old stone mortars at Raqefet Cave, Israel: Investigating Natufian ritual feasting. *Journal of Archaeological Science: Reports* **21**, 783–793 (2018).

35. García-Granero, J. J., Urem-Kotsou, D., Bogaard, A., Kotsos, S. Cooking plant foods in the northern Aegean: Microbotanical evidence from Neolithic Stavroupoli (Thessaloniki, Greece). *Quat. Int.* **496**, 140–151 (2018).

36. Henry, A. G. "Starch Granules as Markers of Diet and Behavior" in *Handbook for the Analysis of Micro-Particles in Archaeological Samples*, A. G. Henry, Ed. (Springer International Publishing, 2020), pp. 97–116.

37. Henry, A. G., Hudson, H. F., Piperno, D. R. Changes in starch grain morphologies from cooking. *J. Archaeol. Sci.* **36**, 915–922 (2009).

38. García-Granero, J. J. *et al.*, Integrating Lipid and Starch Grain Analyses From Pottery Vessels to Explore Prehistoric Foodways in Northern Gujarat, India. *Frontiers in Ecology and Evolution* **10** (2022).

39. Reyes, F. G. R., D'Appolonia, B. L., Ciacco, C. F., Montgomery, M. W. Characterization of Starch from Ginger Root (Zingiber officinale). *Starke* **34**, 40–44 (1982).

40. Reichert, E. T. *The differentiation and specificity of starches in relation to genera, species, etc.; stereochemistry applied to protoplasmic processes and products, and as a strictly scientific basis for the classification of plants and animals* (Carnegie Institution of Washington, 1913).

41. Yu, X., Zhang, J., Li, A., Wang, Z., Xiong, F. Morphology and Physicochemical Properties of 3*Lilium*Bulb Starches. *Journal of Food Science* **80**, C1661–C1669 (2015).

42. Liu, L., Kealhofer, L., Chen, X., Ji, P. A broad-spectrum subsistence economy in Neolithic Inner Mongolia, China: Evidence from grinding stones. *Holocene* **24**, 726–742 (2014).

43. Li, W. *et al.*, Pottery Use and Starchy Foods During the Shuangdun Culture (ca.7.3–6.8 Ka BP) in the Middle Catchment of the Huai River, China. *Frontiers in Earth Science* **10** (2022).

44. Cagnato, C., Hamon, C., Salavert, A., Elliott, M. Developing a Reference Collection for Starch Grain Analysis in Early Neolithic Western Temperate Europe. *Open Archaeology* **7**, 1035–1053 (2021).

45. Cristiani, E. *et al.*, Wild cereal grain consumption among Early Holocene foragers of the Balkans predates the arrival of agriculture. *Elife* **10** (2021).

46. Li, Z., Barton, H., Wang, W., Yang, X. Description of Starch Granules From Edible Acorns (Oak), Palms, and Cycads in Southern China. *Front Earth Sci. Chin.* **10**, 815351 (2022).
